# Quantification of Lipophilicity of 1,2,4-Triazoles Using Micellar Chromatography

**DOI:** 10.1007/s10337-012-2227-3

**Published:** 2012-04-08

**Authors:** Małgorzata Janicka, Katarzyna Stępnik, Anna Pachuta-Stec

**Affiliations:** 1 Department of Physical Chemistry, Faculty of Chemistry, Maria Curie-Skłodowska University, Maria Curie-Skłodowska Sq. 3, 20-031 Lublin, Poland; 2 Department of Organic Chemistry, Faculty of Pharmacy, Medical University, 6 Staszica St, 20-081 Lublin, Poland

**Keywords:** Micellar chromatography, Lipophilicity, Triazoles, log *P*, PCA

## Abstract

High-performance liquid chromatography (HPLC), over-pressured-layer chromatography (OPLC) and thin-layer chromatography (TLC) techniques with micellar mobile phases were proposed to evaluate the lipophilicity of 21 newly synthesized 1,2,4-triazoles, compounds of potential importance in medicine or agriculture as fungicides. Micellar parameters log *k*_*m*_ were compared with extrapolated *R*_*M*0_ values determined from reversed-phase (RP) TLC experimental data obtained on RP-8 stationary phases as well as with log *P* values (Alog *Ps*, AClog *P*, Alog *P*, Mlog *P*, KowWin, xlog *P*2 and xlog *P*3) calculated from molecular structures of solutes tested. The results obtained by applying principal component analysis (PCA) and linear regression showed considerable similarity between partition and retention parameters as alternative lipophilicity descriptors, and indicated micellar chromatography as a suitable technique to study lipophilic properties of organic substances. In micellar HPLC, RP-8e column (Purospher) was applied, whereas in OPLC and TLC, RP-CN plates were applied, which was the novelty of this study and allowed the use of micellar effluents in planar chromatography measurements.

## Introduction

For many years, continued interest in new bioactive compounds for applications in medicine and agriculture has been observed [1–8]. Physicochemical properties of xenobiotics such as solubility, lipophilicity (hydrophobicity), stability and acid–base character affecting absorption, distribution and transport in biological systems should be determined in the early stages of development. The hydrophobic effect is assumed to be one of the driving forces for passive transport of xenobiotics through bio-membranes and, to a certain degree, responsible for interactions with receptors. This property determining the biological activity of substances was first recognized by Overton, Meyer and Baum [2, 4], and since that time hundreds of articles, among them some review papers, on the lipophilic properties of different bioactive compounds in medicine, agriculture or environmental chemistry have appeared [9–16].

Lipophilicity is characterized by solute distribution in biphasic liquid system, and its universal scale is represented by the logarithms of the partition coefficients (log *P*) in the case of neutral species or the distribution ratio (log *D*) for ionisable compounds [12, 17]. In the early 1970s, octanol–water was proposed as a reference system for lipophilicity measurements and to this day remains as a standard for experimental and theoretical investigations. Due to experimental limitations connected with direct measurements of log *P* (log *D*) parameters by shake-flask method, chromatographic techniques are becoming increasingly popular for studying the lipophilic properties of different compounds. Though partition parameters reflect the universal scale of lipophilicity, the chromatographic approach is much more convenient, reproducible, fast and inexpensive. Both types of parameter, i.e. partitioning and chromatographic, are now standardized and officially recommended by the Organization of Economic Co-operation and Development (Guidelines for the Testing of Chemicals).

Although reversed-phase liquid chromatography is most frequently used in studying lipophilicity of xenobiotics, recently new stationary phases imitating biosystems, such as immobilized artificial membranes (IAMs), immobilized proteins [7, 10], ceramides [18], keratin [19] or cholesterol [20, 21], or alternative techniques such as counter-current chromatography (CCC) [22, 23] or micellar liquid chromatography (MLC) [24–32] have been proposed for this purpose.

A universal and widely accepted chromatographic lipophilicity descriptor is the retention factor evaluated by RP LC in the system with water as the mobile phase: log *k*_*w*_ in column or *R*_*M*0_ in planar techniques. This value can be calculated from the Soczewiński–Wachtmeister equation [33]:

1 $$ R_{M} = R_{M0} - s\varphi, $$

where *φ* is the volume fraction of organic modifier in the mobile phase, and *R*_M_ and *R*_*M*0_ are retardation parameters corresponding to mixed effluent or water as the mobile phase, respectively. The regression slope *s* is regarded as a characteristic of the specific hydrophobic area of the solute.

Micellar liquid chromatography is a mode of conventional RP LC using a surfactant solution above the critical micellization concentration (cmc) as the mobile phase [34, 35]. The presence of micelles in the mobile phase is the source of different molecular interactions: solute association with the polar head of the surfactant, solute penetration into the micelle core, adsorption of surfactant monomers on the alkyl-bounded stationary phases as a result of hydrophobic interactions between surfactant tail and alkyl chain, and solute interactions with adsorbed surfactant and alkyl chains. In such systems, solute retention is governed by three different equilibria: solute distribution between the micelles and the bulk phase, solute partition between the stationary phase and the bulk phase, and direct transfer of solute molecules between surfactant-modified surface and the micelles. The latter equilibrium is significant in the case of highly non-polar solutes. Because molecular interactions involving solute depend on its lipophilicity, micellar retention parameters can be considered as lipophilicity descriptors.

According to Foley, there is the following relationship between retention parameter *k* and surfactant concentration in the effluent [36]:

2 $$ \frac{1}{k} = \frac{1}{{k_{m} }} + \frac{{K_{AM} }}{{k_{m} }}[M], $$

where [*M*] is the total concentration of surfactant in the mobile phase, *K*_*AM*_ is the constant describing solute–micelle binding and *k*_*m*_ is the solute retention parameter at zero micellar concentration, i.e. at surfactant monomer concentration equal to the cmc. The parameters *K*_*AM*_ and *k*_*m*_ can be evaluated from the slope and intercept of experimental 1/*k* versus [*M*] relationships. This equation is valid for aqueous solutions of surfactant or mobile phases with the same organic modifier concentrations [34].

The micellar log *k*_*m*_ parameter is considered analogous to log *k*_*w*_ (*R*_*M*0_) evaluated in reversed-phase chromatography and, as a lipophilicity descriptor, correlated with log *P* values. Various workers applying MLC in lipophilicity studies using different substances [24, 29, 33, 34] observed linear relationships between micellar and partitioning or chromatographic lipophilicity parameters [29, 37–39], while another reported the curvature of log *k* versus log *P* plots [26, 40, 41].

In our research, a group of 21 newly synthesized 1,2,4-triazoles [42, 43], potential antifungal compounds currently being tested for biological activity, were examined for lipophilic properties by liquid chromatography. The advantage of the research method presented herein is the use of planar techniques, TLC and OPLC, with micellar mobile phases. So far, micellar effluents, in contrary to column, have rather rarely been applied in planar chromatography, and there is a lack of reports on this topic. Available articles [30, 44–47] relate to fundamental research and not specific applications. In our previous studies [31], newly synthesized *N*-phenyltrichloroacetamide derivatives were investigated for lipophilic properties using micellar TLC and OPLC techniques on RP-18W stationary phases, while in the present research, RP-CN plates were applied.

## Experimental

### Reagents and Materials

The structures of tested 1,2,4-triazoles, synthesized in our laboratory, are presented in Table 1. Sodium dodecyl sulphate (SDS) (for synthesis), tetrahydrofuran and acetonitrile (both of HPLC grade) as well as chromatographic plates RP-CN F_254s_ and RP-8 F_254s_ (10 × 10 cm) were purchased from Merck. Citric acid and Na_2_HPO_4_ (both pure) were supplied from POCh. Distilled water was obtained from Direct-Q 3 UV apparatus (Millipore).

**Table 1:** Structures, computed log *P* and log *k*_*m*_ values of tested compounds

No.	R–	Alog *Ps*	AClog *P*	Alog *P*	Mlog *P*	KowWin	xlog *P*2	xlog *P*3	log *P*_aver._	log *k*_*m*_,_HPLC_	log *k*_*m*_,_OPLC_	log *k*_*m*_,_TLC_

1	CH_3_–CH_2_–CH_2_–	2.46	2.11	2.68	2.80	2.58	2.56	2.67	2.55 ± 0.25	1.00	0.73	0.78
2	CH_3_–CH_2_–CH_2_CH_2_–	3.02	2.52	3.05	3.05	3.00	3.02	3.10	2.97 ± 0.21	1.23	0.82	0.88
3	CH_3_–CH_2_–CH_2_–CH_2_–	2.79	2.58	3.13	3.06	3.07	3.13	3.02	2.97 ± 0.20	1.31	0.88	0.93
4	C_6_H_5_–CH_2_–	2.64	2.58	3.39	3.55	3.30	3.49	3.27	3.17 ± 0.40	1.46	0.93	1.02
5	C_6_H_5_–	3.26	2.79	3.38	3.57	3.39	3.35	3.33	3.30 ± 0.24	1.48	0.97	1.09
6	4-CH_3_–O–C_6_H_4_–	2.96	2.69	3.36	3.31	3.47	3.26	3.30	3.19 ± 0.27	1.50	0.93	1.02
7	Cyclohexyl-	3.27	2.80	3.66	3.57	3.86	3.61	3.58	3.48 ± 0.35	1.68	1.02	1.11
8	C_6_H_5_–CH_2_–CH_2_–	3.00	2.92	3.71	3.79	3.79	3.65	3.73	3.51 ± 0.38	1.80	1.05	1.16
9	2-Cl–C_6_H_4_–	3.94	3.41	4.04	4.09	3.47	3.97	3.96	3.84 ± 0.20	1.88	1.21	1.22
10	4-Br–C_6_H_4_–	3.87	3.49	4.13	4.20	4.28	4.15	4.02	4.02 ± 0.27	2.22	1.23	1.36

11	CH_3_–CH_2_–CH_2_–	1.64	1.84	1.91	2.97	2.25	2.14	2.15	2.13 ± 0.43	0.85	0.65	0.69
12	CH_3_–CH_2_–CH_2_CH_2_–	2.37	2.24	2.28	3.22	2.67	2.60	2.58	2.57 ± 0.33	1.12	0.75	0.82
13	CH_3_–CH_2_–CH_2_–CH_2_–	2.01	2.30	2.36	3.22	2.74	2.71	2.51	2.55 ± 0.39	1.20	0.83	0.89
14	C_6_H_5_–CH_2_–	2.15	2.31	2.62	3.71	2.98	3.07	2.75	2.80 ± 0.52	1.33	0.86	0.92
15	C_6_H_5_–	2.68	2.52	2.61	3.74	3.07	2.93	2.81	2.91 ± 0.41	1.32	0.92	0.96
16	4-CH_3_–O–C_6_H_4_–	2.78	2.41	2.59	3.47	3.15	2.84	2.79	2.86 ± 0.35	1.27	0.81	0.87
17	Cyclohexyl-	2.30	2.52	2.89	3.72	3.54	3.19	3.07	3.03 ± 0.51	1.57	0.95	1.03
18	C_6_H_5_–CH_2_–CH_2_–	2.40	2.65	2.94	3.94	3.47	3.23	3.21	3.12 ± 0.51	1.68	1.01	1.11
19	2-Cl–C_6_H_4_–	3.27	3.13	3.28	4.24	3.15	3.55	3.44	3.44 ± 0.39	1.79	1.07	1.13
20	4-Br–C_6_H_4_–	3.58	3.21	3.36	4.36	3.96	3.73	3.51	3.67 ± 0.36	2.00	1.14	1.25
21	3-CH_3_–C_6_H_4_–	2.95	2.83	3.10	3.97	3.61	3.37	3.18	3.29 ± 0.40	1.66	0.98	1.08

### Chromatographic Measurements

#### Micellar HPLC

A Shimadzu Vp liquid chromatographic system equipped with LC 10AT pump, SPD 10A UV–VIS detector, SCL 10A system controller, CTO-10 AS chromatographic oven and Rheodyne injector valve with a 20-μL loop was applied in HPLC measurements. The stainless-steel RP-8e column (Purospher, 12.5 cm × 4 mm, i.d., 5 μm particle size) was used as stationary phase. All measurements were carried out at 20 °C at flow rate of 1.3 mL min^−1^. The tested compounds, separately dissolved in acetonitrile (about 0.01 mg mL^−1^), were detected under ultraviolet (UV) light at 230 nm. Mobile phases were composed of 0.04, 0.06, 0.08 and 0.1 M SDS in buffer (0.01 M Na_2_HPO_4_/0.01 M citric acid) with 20 % addition of acetonitrile. The dead time values (*t*_0_), measured from solvent peak, were as follows: *t*_0_(0.04 M SDS) = 32.49 s, *t*_0_(0.06 M SDS) = 32.17 s, *t*_0_(0.08 M SDS) = 32.49 s and *t*_0_(0.1 M SDS) = 32.32 s. For calculation of retention factors, average values from at least three experimental data were used.

#### Micellar TLC and OPLC

Sandwich chambers (Chromdes, Poland) used in TLC measurements were saturated with organic modifier of the mobile phase for 15 min before development. In OPLC experiments, OPLC BS 50 chamber (OPLC-NIT, Hungary) in fully off-line mode [48, 49] was used with the following operating conditions: *V*_r_ = 200 μL, *V*_e_ = 600–700 μL, *u* = 200 μL min^−1^. The substances were dissolved in methanol (0.1 mg mL^−1^), and 1-μL volumes were applied on the plates by a microsyringe. As stationary phase, RP-CN F_254s_ plates were used. In micellar TLC, application of octadecylsilyl (ODS)-type stationary phases as usually used in lipophilicity studies is problematic. Water-rich micellar effluents hardly wet RP-18 or RP-8 phases, which increases so-called thin-layer effects such as mobile phase demixing or phase gradient formation. The application of RP-CN stationary phases not only facilitates chromatographic system equilibration but also reduces the analysis time. As mobile phases, solutions of 0.03, 0.04, 0.06, 0.08 and 0.1 M SDS in buffer were used, modified by constant (20 %, v/v) addition of tetrahydrofuran. Solutes no. 1–4, 7, 8, 11–14 and 21 were detected in UV light at 200 nm by the use of a Shimadzu scanner Cs-9000, and the others at 254 nm by means of a Reprostar 3 video camera and video scan (CAMAG). Each value was determined in duplicate.

#### Reversed-Phase TLC

TLC RP-8 F_254s_ plates were applied as stationary phases. Buffered solutions of acetonitrile and tetrahydrofuran (organic modifiers used in micellar effluents) were used as effluents. Organic solvent concentration, expressed as volume fraction v/v, varied in the range from 0.3 to 0.7, in constant steps of 0.1. All other stages of experiments (application of solutes, development of plates and detection of solutes) were the same as in the micellar TLC technique.

Physiological pH (7.4) of the buffer was fixed before mixing with organic modifier. Micellar mobile phases were filtered through 0.45-μm membrane filter before use.

In micellar and reversed-phase chromatography, the following systems were applied:

- Micellar HPLC: RP-8e/buffered SDS—acetonitrile (4:1, v/v)
- Micellar OPLC: RP-CN/buffered SDS—tetrahydrofuran (4:1, v/v)
- Micellar TLC: RP-CN/buffered SDS—tetrahydrofuran (4:1, v/v)
- RP TLC1: RP-8/buffer—acetonitrile
- RP TLC2: RP-8/buffer—tetrahydrofuran

Statistical calculations were performed using Minitab 16 software.

## Results and Discussion

### Computed log *P* Parameters

**Fig. 1:**
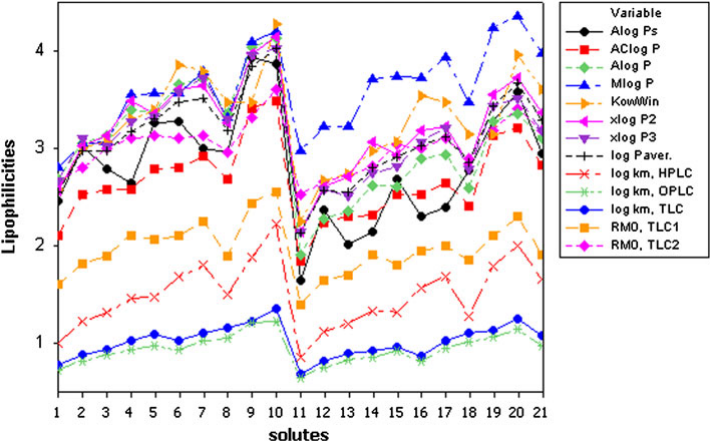
Lipophilicity profiles of investigated solutes

Partition coefficients log *P*, calculated according to molecular structures by use of program packages available at the Virtual Computational Chemistry Laboratory as described in the literature [50, 51], are summarized in Table 1. The calculations of log *P* values are based on well-characterized log *P* contributions of separate atoms, structural fragments and intramolecular interactions between different fragments (Alog *Ps*, AClog *P*, KowWin, xlog *P*2 and xlog *P*3) or molecular descriptors (Alog *P*, Mlog *P*) [51]. Lipophilicity profiles shown in Fig. 1 demonstrate certain discrepancies for particular log *P* values, i.e. Alog *Ps*, KowWin or Mlog *P*. The eigenvalues obtained by applying PCA (Table 2) show that the first principal component accounts for 88.5 % only, while the first three components account for 98.1 %. The results strengthen doubts in relation to computed log *P* values as accurate lipophilicity descriptors, and it seems interesting and reasonable to compare them with experimental chromatographic indices.

**Table 2:** Eigenanalysis of the correlation matrix of computed log *P* and chromatographic log *k*_*m*_ and *R*_*M*0_ parameters

Principal component	PCA of log *P* values		PCA of log *P*, log *k*_*m*_, *R*_*M*0_ values	
	Eigenvalue	Cumulative proportion (%)	Eigenvalue	Cumulative proportion (%)
1	7.0784	88.5	11.606	89.3
2	0.5221	95.0	0.596	93.9
3	0.2511	98.1	0.355	96.6
4	0.1086	99.5	0.186	98.0
5	0.0197	99.8	0.115	98.9
6	0.0166	100.0	0.054	99.3
7	0.0034	100.0	0.037	99.6
8	0.0000	100.0	0.027	99.8
9	–	–	0.013	99.9
10	–	–	0.007	100.0
11	–	–	0.003	100.0
12	–	–	0.001	100.0
13	–	–	0.000	100.0

### Chromatographic Lipophilicity Parameters (*R*_*M*0_, log *k*_m_)

For all solutes, regardless of the chromatographic system, linear relationships corresponding to Eqs. (1) and (2) were obtained (see *R* ^2^ values in Table 3); *R*_*M*0_ and log *k*_*m*_ values calculated from these relationships are summarized in Tables 1 and 3. Parallel lipophilicity profiles illustrated in Fig. 1 indicate high correlations between chromatographic *R*_*M*0_ and log *k*_*m*_ values and computed log *P* parameters. Both chromatographic and partitioning lipophilicity indices show the same effect of solute structure on lipophilicity. Compounds of type A are more lipophilic than those of type B, indicating the hydrocarbon ring as the decisive factor affecting lipophilicity. Regular, almost linear, increase of lipophilic properties of solutes no. 1–3 or 11–13 and no. 8–10 or 18–20 corresponds to the increase of lipophilic character with substitution of the secondary amine group. Micellar log *k*_*m*_ parameters are visibly lower than *R*_*M*0_ or computed log *P* values, undoubtedly as a result of addition of an organic modifier to the micellar mobile phase.

**Table 3:** Parameters of Eq. (1) (RP TLC1, RP TLC2) and Eq. (2) (micellar HPLC, micellar OPLC, micellar TLC) calculated for solutes tested

Solute	RP TLC1			RP TLC2			Micellar HPLC			Micellar OPLC			Micellar TLC		
	*R*_*M*0_	*s*	*R* ^2^	*R*_*M*0_	*s*	*R* ^2^	$\frac{{K_{AM} }}{{k_{m} }}$	$\frac{1}{{k_{m} }}$	*R* ^2^	$\frac{{K_{AM} }}{{k_{m} }}$	$\frac{1}{{k_{m} }}$	*R* ^2^	$\frac{{K_{AM} }}{{k_{m} }}$	$\frac{1}{{k_{m} }}$	*R* ^2^
1	1.60	3.06	0.977	2.66	4.59	0.976	1.344	0.100	0.973	0.156	0.186	0.893	0.156	0.166	0.942
2	1.82	3.36	0.985	2.80	4.75	0.982	0.825	0.059	0.988	0.313	0.151	0.921	0.219	0.131	0.993
3	1.90	3.46	0.966	3.00	4.99	0.987	0.813	0.049	0.991	0.496	0.132	0.987	0.125	0.117	0.983
4	2.11	3.72	0.982	3.10	5.12	0.988	0.781	0.035	0.989	0.281	0.117	0.932	0.688	0.095	0.977
5	2.06	3.76	0.971	3.13	5.29	0.978	0.772	0.033	0.997	2.969	0.107	0.972	1.500	0.081	0.964
6	2.10	3.74	0.919	3.10	5.10	0.987	0.843	0.031	0.999	0.300	0.118	0.835	2.106	0.096	0.962
7	2.25	3.90	0.963	3.13	5.15	0.982	0.488	0.021	0.994	0.781	0.095	0.869	0.844	0.078	0.948
8	1.90	3.50	0.955	2.96	5.00	0.985	0.619	0.016	0.993	0.875	0.090	0.904	1.219	0.069	0.940
9	2.44	4.28	0.987	3.32	5.42	0.989	0.563	0.013	0.996	3.500	0.062	0.977	4.063	0.060	0.807
10	2.55	4.20	0.975	3.60	5.78	0.990	0.525	0.006	0.997	2.656	0.059	0.996	2.549	0.044	0.927
11	1.40	2.79	0.976	2.52	4.42	0.988	1.113	0.140	0.964	0.313	0.224	0.987	1.250	0.205	0.924
12	1.65	3.13	0.982	2.66	4.59	0.979	0.806	0.075	0.990	0.375	0.178	0.991	0.875	0.150	0.998
13	1.70	3.18	0.993	2.72	4.65	0.969	0.744	0.063	0.988	0.625	0.148	0.985	0.438	0.129	0.954
14	1.91	3.48	0.986	2.81	4.77	0.989	0.681	0.047	0.986	0.281	0.138	0.992	0.938	0.120	0.927
15	1.80	3.23	0.981	3.04	5.03	0.989	0.725	0.048	0.982	4.031	0.120	1.000	1.406	0.110	0.996
16	1.95	3.52	0.979	3.00	5.01	0.990	0.831	0.054	0.988	3.563	0.155	0.940	0.061	0.136	1.000
17	2.00	3.58	0.983	3.10	5.05	0.979	0.506	0.027	0.998	0.469	0.113	0.932	0.750	0.093	0.991
18	1.86	3.39	0.989	2.89	4.79	0.976	0.550	0.021	0.995	0.344	0.098	0.989	0.594	0.078	0.990
19	2.10	3.80	0.989	3.19	5.20	0.985	0.800	0.016	0.936	3.688	0.085	1.000	1.570	0.074	0.892
20	2.30	3.99	0.985	3.41	5.30	0.991	0.463	0.010	0.996	3.250	0.072	0.997	1.406	0.056	0.996
21	1.91	3.42	0.991	3.18	4.96	0.989	0.550	0.022	0.995	2.156	0.105	0.997	1.094	0.084	0.993

**Fig. 2:**
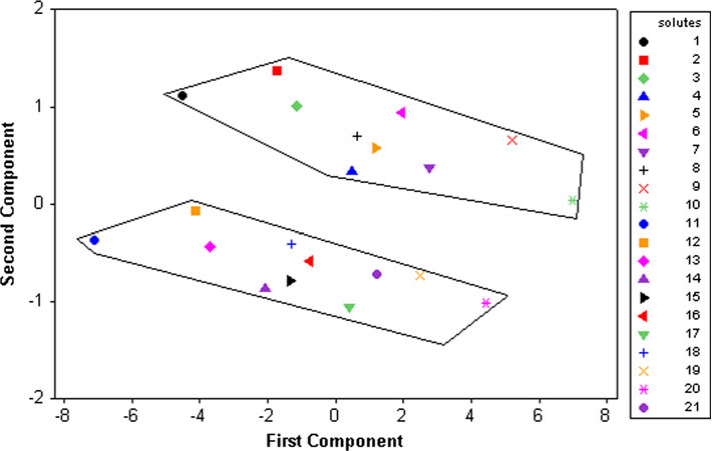
Score plot of log *P* log *k*_*m*_ and *R*_*M*0_ values

PCA was applied to compare computed log *P* and chromatographic (*R*_*M*0_, log *k*_*m*_) parameters, and the results show that the first three components account for 96.6 % (Table 2). The score plot presented in Fig. 2 demonstrates the similarities and dissimilarities between tested substances according to log *P*, log *k*_*m*_ and *R*_*M*0_ values evaluated from different systems: two separate clusters corresponding to solutes with structures of type A and B are formed.

Detailed evaluation of micellar log *k*_*m*_ parameters as lipophilicity descriptors was carried out by comparing them with partitioning log *P* or *R*_*M*0_ values, using linear regression. For this purpose, Collander-type equations [2], i.e. direct linear correlations between log *P* and log *k*_*m*_ or *R*_*M*0_ values, were analysed, and the best results are presented in Table 4. In these studies, separate relationships for two groups of solutes tested were obtained. The best linearity was observed between micellar parameters and xlog *P*2, xlog *P*3 and log *P*_aver._ values, as for HPLC, OPLC and TLC techniques. Analogous relationships corresponding to *R*_*M*0_ values and characterized by much lower coefficients of determination demonstrate that extrapolated *R*_*M*0_ parameters rather poorly correlate with partitioning lipophilicity descriptors.

**Table 4:** Correlation matrix for various log *P* versus log *k*_*m*_ or log *P* versus *R*_*M*0_ relationships

Relationships	Solutes no. 1–10		Solutes no. 11–21	
	*R* ^2^	Residual mean^2^	*R* ^2^	Residual mean^2^
xlog *P*2 versus log *k*_*m*,HPLC_	0.965	0.007	0.961	0.008
xlog *P*2 versus log *k*_*m*,OPLC_	0.980	0.004	0.936	0.014
xlog *P*2 versus log *k*_*m*,TLC_	0.972	0.006	0.938	0.013
xlog *P*2 versus *R*_*M*0,TLC1_	0.833	0.035	0.867	0.028
xlog *P*2 versus *R*_*M*0,TLC2_	0.813	0.040	0.897	0.022
xlog *P*3 versus log *k*_*m*,HPLC_	0.944	0.014	0.965	0.008
xlog *P*3 versus log *k*_*m*,OPLC_	0.954	0.011	0.945	0.013
xlog *P*3 versus log *k*_*m*,TLC_	0.947	0.013	0.946	0.012
xlog *P*3 versus *R*_*M*0,TLC1_	0.839	0.039	0.875	0.029
xlog *P*3 versus *R*_*M*0,TLC2_	0.719	0.044	0.826	0.040
log *P*_aver._ versus log *k*_*m*,HPLC_	0.949	0.010	0.974	0.005
log *P*_aver._ versus log *k*_*m*,OPLC_	0.959	0.008	0.940	0.012
log *P*_aver._ versus log *k*_*m*,TLC_	0.940	0.012	0.945	0.011
log *P*_aver._ versus *R*_*M*0,TLC1_	0.751	0.051	0.822	0.034
log *P*_aver._ versus *R*_*M*0,TLC2_	0.705	0.060	0.817	0.035

## Conclusions

In this work, reversed-phase TLC and micellar HPLC, OPLC and TLC were used to examine a group of 21 newly synthesized 1,2,4-triazoles. Lipophilic properties of substances tested were characterized by micellar log *k*_*m*_, reversed-phase *R*_*M*0_ and computed log *P* values. Similarities between lipophilicity indices were analysed by PCA and linear regression. Highly significant correlations obtained between computed log *P*, especially xlog *P*2, xlog *P*3 and log *P*_aver._ and log *k*_*m*_ values show micellar chromatography to be an excellent technique for studying lipophilicity of triazoles. Moreover, application of RP-CN stationary phases allowed use of micellar effluents in planar chromatography (TLC and OPLC) measurements. In this work, OPLC seems to be an especially suitable technique due to the significant reduction in reagent consumption and analysis time.
